# How much do we know about the metastatic process?

**DOI:** 10.1007/s10585-023-10248-0

**Published:** 2024-03-23

**Authors:** Carolina Rodriguez-Tirado, Maria Soledad Sosa

**Affiliations:** 1 Department of Microbiology and Immunology, Albert Einstein College of Medicine/Montefiore Medical Center, Bronx, NY 10461 USA; 2 Department of Oncology, Albert Einstein College of Medicine/Montefiore Medical Center, Bronx, NY 10461 USA; 3 Montefiore Einstein Comprehensive Cancer Center, Albert Einstein College of Medicine/Montefiore Medical Center, Bronx, NY 10461 USA; 4 Cancer Dormancy and Tumor Microenvironment Institute/Montefiore Medical Center, Albert Einstein College of Medicine, Bronx, NY 10461 USA; 5 Ruth L. and David S. Gottesman Institute for Stem Cell Research and Regenerative Medicine, Albert Einstein College of Medicine/Montefiore Medical Center, Bronx, NY 10461 USA

**Keywords:** Early dissemination, Disseminated cancer cells, Dormancy, Metastasis, EMT

## Abstract

Cancer cells can leave their primary sites and travel through the circulation to distant sites, where they lodge as disseminated cancer cells (DCCs), even during the early and asymptomatic stages of tumor progression. In experimental models and clinical samples, DCCs can be detected in a non-proliferative state, defined as cellular dormancy. This state can persist for extended periods until DCCs reawaken, usually in response to niche-derived reactivation signals. Therefore, their clinical detection in sites like lymph nodes and bone marrow is linked to poor survival. Current cancer therapy designs are based on the biology of the primary tumor and do not target the biology of the dormant DCC population and thus fail to eradicate the initial or subsequent waves of metastasis. In this brief review, we discuss the current methods for detecting DCCs and highlight new strategies that aim to target DCCs that constitute minimal residual disease to reduce or prevent metastasis formation. Furthermore, we present current evidence on the relevance of DCCs derived from early stages of tumor progression in metastatic disease and describe the animal models available for their study. We also discuss our current understanding of the dissemination mechanisms utilized by genetically less- and more-advanced cancer cells, which include the functional analysis of intermediate or hybrid states of epithelial–mesenchymal transition (EMT). Finally, we raise some intriguing questions regarding the clinical impact of studying the crosstalk between evolutionary waves of DCCs and the initiation of metastatic disease.

## Introduction

**Fig. 1:**
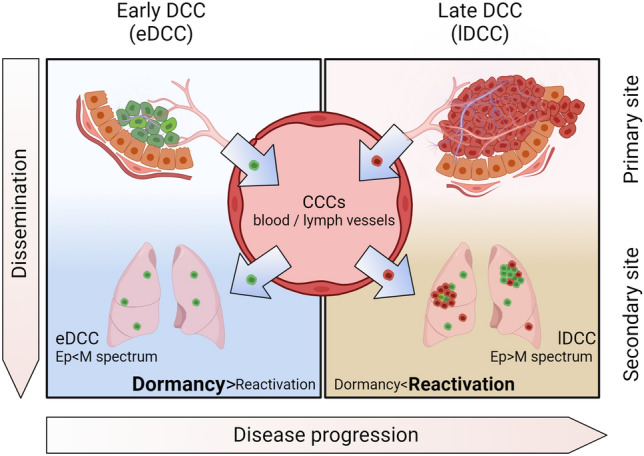
Dissemination of cancer cells. Cancer cells disseminate in waves throughout primary tumor evolution, from early stages (early dissemination, green cells) to late stages (late dissemination, red cells), through blood or lymphatic vessels. While in circulation, circulating cancer cells (CCCs) display a spectrum of epithelial (Ep)/mesenchymal (M) phenotypes that are associated with aggressiveness, chemotherapy resistance, and survival, which appears to be maintained by cancer cells after arrival at secondary organs (see section entitled “Partial EMT is linked to dissemination and metastatic colonization”). Generally, DCCs remain as single cells after activating a cellular dormancy program. As shown in breast cancer mouse models, during the early stages of tumor evolution, the lungs of animals harbor a considerable number of early DCCs (eDCCs, green cells in the lungs) characterized by mesenchymal and hybrid phenotypes [12]. Although eDCCs are capable of reactivation, they remain as dormant single cells for prolonged periods [10–12], suggesting that the required signals to escape dormancy are not present or not sufficiently abundant at this stage of the disease. Gradually, the primary tumor evolves into a genetically advanced lesion (red primary tumor), from which late DCCs or lDCCs disseminate (red cells in the lungs). Through the circulation, lDCCs reach secondary organs that have already been colonized by eDCCs, where they can potentially interact. Subsequently, the reactivation signals reach a sufficient level to stimulate DCCs to exit from dormancy and development of metastatic tumors. Clinical [19–23] and experimental evidence [10–12] suggest that in certain types of tumors, metastases arise from eDCCs (parallel dissemination [9], metastasis with a majority of cells in green). (Color figure online)

For decades, the hallmarks of cancer scheme [1] has helped the cancer community to describe the multistep development of human tumors including metastatic dissemination. A variable difficult to represent in the hallmarks of cancer’s scheme [1] is *time*, i.e., the duration of each event (and what controls this duration, particularly after cancer cells have reached distant sites), and when metastatic dissemination occurs. For example, while the development of a primary tumor can take a few years to decades, intravasation and dissemination through the blood system can occur within minutes or hours. Moreover, after disseminated cancer cells (DCCs) lodge at distant sites, they can remain undetected for years or decades by entering a non-proliferative state defined as *cellular dormancy* (BOX 1 [2–6]). A recent review addressed the definition and mechanisms of cellular dormancy [7]. Eventually, DCCs that exit this dormancy state will form life-threatening metastases. For many years, we have accepted the notion that cancer progression follows a *linear model* whereby the cumulative acquisition of genetic and epigenetic alterations allows fully malignant cells from advanced stages to spread and metastasize. Over the last 20 years, the concept of *parallel progression* of primary tumors and metastases has gained traction throughout the scientific community. The parallel progression model posits that cancer cells, presumably with fewer genetic alterations, can disseminate to distant sites before developing a fully malignant phenotype at the primary site. With time, these cells can accumulate independent genetic and epigenetic alterations at distant sites [8], ultimately resulting in metastasis development [9]. In this review, we discuss several lines of evidence that support the early timing of metastatic dissemination. For instance, an experimental breast cancer mouse model revealed that early DCCs (eDCCs) originating from genetically less-advanced cancer cells (e.g., cancer cells found in hyperplastic lesions that resemble human ductal carcinoma in situ (DCIS)) also undergo dormancy and might, under the right conditions, establish metastases with time [10–12] (Fig. 1). In clinical settings, 14–31% of patients with DCIS lesions, classified as non-invasive early lesions, have detectable DCCs in the bone marrow compartment [13–18], arguing that these pre-malignant cells have the capacity to disseminate. Notably, a small percentage of patients with DCIS die of breast metastases 15–20 years later without experiencing the formation of an invasive carcinoma [19, 20], suggesting that eDCCs derived from the DCIS lesion can lead to metastasis formation after an extended dormancy phase.

An extensive body of literature has dissected (and continues to do so) the mechanisms and signaling pathways relevant to disease progression in cancers of multiple origins, although it mainly focuses on primary sites. Although these investigations have described the milestones needed for the discovery and design of therapies that have significantly improved the life expectancy and quality of life of patients for decades, such as paclitaxel and trastuzumab [24], it is undeniable that current therapies frequently fail to treat metastatic disease. One reason for this is that the design of current treatments does not take into consideration the biology of dormant DCC subpopulations and thus fail to eradicate metastases, which are responsible for most deaths in patients diagnosed with solid tumors [25]. Therefore, it is reasonable to investigate whether targeting DCC subpopulations would yield any advantage in treating metastasis. To accomplish this goal, we need to overcome the limitations from our poor understanding of DCC biology compared to tumor mass biology.

Incorporating these emerging crucial concepts (cancer cell dormancy and the parallel progression model) in the study of metastatic disease gives rise to key questions: Do eDCCs (derived from the early stages of tumor progression) survive long enough at distant sites to be solely responsible for relapses in secondary organs? In patients with invasive stages, do eDCCs interact with more genetically evolved DCCs derived from fully developed primary tumors? Which DCC subpopulation (derived from early lesions or genetically evolved primary tumors) is lethal? These questions are critical, as they could possibly explain and more accurately predict the timing of metastasis. Likewise, the time DCCs spend in a non-proliferative dormant state and/or, for any reason, fail to develop into metastatic lesions provides a significant temporal window for therapeutic intervention that the oncology community has largely underutilized. Here we provide a perspective on how DCC biology, including eDCCs spreading during early stages of tumor progression, may lead to a new understanding of the metastatic process.

## Models of cancer dissemination

### Early metastatic dissemination

The parallel model of tumor progression was proposed based on previous efforts to measure human cancer growth rates and suggested that metastasis must be initiated before a primary tumor is detectable [26, 27]. The reason behind this idea was that considering similar growth rates for primary tumors and metastases then the size of the metastases at diagnosis were too large if they originated from the late stages of the primary tumor. This model challenges the widely accepted linear model of progression [9, 28] and has brought into question when metastatic dissemination occurs. Historically, it has been inferred from histopathological observations that cancer cells acquire invasive properties at the late stages of tumor development [29]; therefore, dissemination must be restricted to this stage onward. However, compelling evidence favoring early dissemination in humans and mouse models has been presented for breast [10, 11, 13, 17, 19, 30–34], melanoma [8, 35–37], colorectal [21, 22], pancreatic [38], lung [39, 40], ovarian [41], and esophageal cancers [42].

For instance, 3.3% of women diagnosed with DCIS, a predominantly non-invasive breast tumor, carry a risk of dying from breast cancer even 15 years after diagnosis, which is greater than that of the general US population [19, 20]. The risk is at least twice as high for women diagnosed before 35 years of age, as well as for black women compared to non-Hispanic white women [19]. Considering that half of the women diagnosed with DCIS who eventually died never developed signs of local recurrence (ipsilateral invasive or contralateral invasive breast cancer), it is probable that their metastatic disease was derived from cancer cells that disseminated from DCIS. Alternatively, it can be argued that clinicians might have missed the invasive front in the DCIS biopsies, given the limited sampling in biopsy procedures. Therefore, improved methods for detecting early spread, including the use of large format sections in histopathology practice, are necessary. As such, new classifications in the DCIS field have been proposed based on an imaging biomarker system (BOX 2) that may help to predict early spread. Several pre-clinical models of breast cancer have supported the idea that cancer cells derived from stages resembling human DCIS could form metastasis [11, 13], and see Table 1 in “Experimental animal models to study early metastatic dissemination” section for more details of the models).

**Table 1:** Experimental animal models to study early metastatic dissemination

Disease modeled	Animal model	Advantages	Disadvantages	Validation	References
Breast cancer	MMTV-HER2^V664E^	Hyperplastic lesions at 7–9 weeks of age Detectable mammary tumors at 14–18 weeks. Large lung metastases found at 20–25 weeks of age. The temporal window to study early dissemination events is about 7 weeks. eDCCs are found in bone marrow. Spontaneous model, widely used, commercially available.	Model for HER2+breast cancer only (relevant for 30% of total breast cancer patients). The model does not spontaneously develop bone metastasis. Only 24% of HER2 + breast cancer patients metastasize to lungs.	Early dissemination events shown. Clonal phylogenetic analysis showed that ~ 80% of metastasis derived from eDCCs. Metastatic potential of eDCCs validated.	[10, 13]
	MMTV-HER2^WT^	Hyperplastic lesions at 16–18 weeks of age. Detectable mammary tumors ~ 30 weeks of age. Longer latency than HER2^V664E^. The temporal window to study early dissemination events is about 15 weeks. Dormant eDCCs are found in lung and bone marrow. Contribution of macrophages to early dissemination events. Detection of TMEM structures. Spontaneous model, widely used, commercially available.	Model for HER2+breast cancer only (relevant for 30% of total breast cancer patients). The model does not spontaneously develop bone metastasis Only 24% of HER2+breast cancer patients metastasize to lungs.	Early dissemination mechanisms described. Metastatic potential of eDCCs validated. Dormancy signature described in eDCCs.	[11, 12, 33, 43]
	MMTV-PyMT	Hyperplasia at 4 weeks of age. Detectable primary tumors at 14 weeks of age. Temporal window to study early dissemination events is about 10 weeks. eDCCs detected at hyperplastic stage in bone marrow and lungs. eDCCs express stem cell markers. Detection of TMEM structures. Spontaneous model, widely used and commercially available.	PyMT is not a human oncogene.	Similarity to Luminal B tumors in humans. Early dissemination demonstrated.	[13, 33, 44–49]
	MIND Xenografts (Mouse IntraDuctal human-in-mouse DCIS model)	Primary cells isolated from patients carrying DCIS and atypical hyperplasia lesions as well as DCIS cell lines maintain cellular characteristics of tissue of origin when injected in immunocompromised host animals. DCIS lesions are maintained for at least 8 weeks. Some DCIS lesions progress to invasive ductal carcinomas. Closely mimics human DCIS, with potential to study early dissemination.	Immunocompromised host animals required. Interaction of DCIS cells with the microenvironment (immune cells, ECM) may not recapitulate fully human tumor biology. Pharmacodynamics in mouse host may be different than in humans. Early dissemination not proven yet.	Model to study early disease that could help predict progressors from non-progressors for patients diagnosed with DCIS.	[19, 50, 51]
		Human cell lines reproduce DCIS-like lesions which progress to invasive carcinomas when injected in host animals: ~ 25% of basal-like DCIS generated by MCF10DCIS.COM cells will progress into invasive cancer in 10 weeks. HER2 + DCIS-like lesions generated by SUM-225 cells will progress into invasive cancer after 14 weeks. Cell lines commercially available with minimal requirements for maintenance and expansion in vitro.	Immunocompromised host animals required. Interaction of DCIS-like cells with the microenvironment may not recapitulate fully human biology. Pharmacodynamics in mouse host may be different than in humans. Tumor heterogeneity might be reduced. Early dissemination not proven yet.	Model to study early disease that could help predict progressors from non-progressors for patients diagnosed with DCIS.	[51, 52]
Pancreatic cancer	KPYC (Kras^G12D^**-**LSL, P53^fl/+^, YFP-LSL, Pdx1-Cre)	Conditional model (floxed P53, Lox-Stop-Lox(LSL)-KRAS^G12D^ and LSL-YFP) mediated by Cre recombinase under Pdx1 promoter. Lineage tracing of all Cre expressing cells with YFP. Mice develop early PanIN at 8–10 weeks of age. Development of PDAC detectable after 16 weeks of age, with metastasis to multiple organs. CCCs and eDCCs in liver detected at PanIN stage before invasive stage.	Model is not commercially available but can be breed from KPC mice (see below).	Early dissemination validated by CCC and DCC detection. The metastatic potential of eDCCs is not shown. Model suitable to study dormancy and reactivation of eDCCs.	[35, 53]
Pancreatic cancer	KPC (**K**RAS^G12D^-LSL, **P**53^fl/+^, Pdx1-**C**re)	Conditional model (floxed P53, Lox-Stop-Lox(LSL)-KRAS^G12D^) expressing Cre recombinase under Pdx1 promoter. Develop early PanIN at 8–10 weeks of age. Development of PDAC varies from 10–30 weeks of age. Resemble human disease, with temporal window to study early dissemination ranging 2–22 weeks. Metastasis to multiple organs. Widely used, commercially available	No lineage tracing available.	Recapitulate different stages of pancreatic PDAC. Dissemination at early stage has not been evaluated by CCC or DCC detection. Metastatic potential of eDCCs has not been evaluated. Dormancy of DCCs has not been evaluated.	[54–57]
	KC (**K**RAS^G12D^-LSL, P48-**C**re)	Conditional model (LSL-KRAS^G12D^) driven by Cre expression under pancreatic promoter (P48) Mice develop PanIN around 4 weeks of age. PDAC detection after 30 weeks of age, with temporal window to study early dissemination events of about 25 weeks. Primary tumor and metastasis onsets vary based on animal backgrounds. Conditional model, widely used, commercially available.	KRAS mutation alone is seldom found in human disease.	Model use to study early events of pancreatic cancer. Dissemination at early stage has not been evaluated by CCC or DTC detection. Metastatic potential of eDCCs has not been evaluated. Dormancy of DCCs has not been evaluated.	[58–61]
Melanoma	RET.ADD	Spontaneous uveal melanoma model DCCs remain dormant. Dissemination occurs from hyperplastic stage. Latency varies based on animal background. Chimeric MHC ADD allows monitoring of CD8 T cells.	Dormancy length depends on the type of organs (Sclera: 2 weeks, Lungs: up to 1 year). Activated version of RET oncogene is not present in humans.	Recapitulates melanoma progression to metastatic stage. Early dissemination is already shown. Immune regulation of dormancy is suggested.	[35, 62, 63]

Importantly, the rates of distant metastasis and survival of women diagnosed with early-stage (I or II) breast cancer were similar to those who underwent radical mastectomy (removal of the entire breast) or lumpectomy (removal of the tumor mass with a narrow margin of normal tissue). This observation opens the possibility that early cancer clones contribute to distant metastasis and, therefore, survival. However, the incidence of local tumor recurrence in the ipsilateral breast was greater in the lumpectomy group than in the mastectomy group [64, 65], arguing that different biology governs local and distant recurrence.

Although it may seem counterintuitive that small in situ-staged tumors disseminate, this is strongly supported by extensive literature [8, 10, 11, 13, 19, 21, 22, 32–35, 38]. For instance, metastatic cancers of unknown primary tumors (or carcinoma of unknown primary (CUP)) may be examples of the early acquisition of a migratory phenotype. Melanoma is the most frequent CUP, and several studies have demonstrated early spread from indolent primary lesions in human samples and in a melanoma mouse model ([8, 35] and see Table 1 in “Experimental animal models to study early metastatic dissemination” section for more details of the model). A recent study described the proposed role of the sodium leak channel non-selective protein (NALCN) as a regulator of cancer cell shedding in different adenocarcinomas as well as normal epithelial cells lacking oncogenic alleles [66]. This suggests the possibility of decoupling dissemination from different stages of tumorigenesis. Whether early cancer lesions could exploit ion channel-dependent mechanisms to spread remains to be evaluated.

Interestingly, patients with DCIS and stage T1 breast cancer have the same number of DCCs in their bone marrow aspirates, suggesting that the DCIS stages are capable of dissemination to the same extent as invasive carcinomas [13]. Moreover, an intriguing study involving patients with invasive breast cancer showed that 10 mm tumors that grow to 90 mm in size exhibit an increase in the rate of distant metastasis ranging from approximately 0.5–26%. In contrast, the contribution of larger tumors (> 60 mm) to distant metastasis plateaued (at approximately 26%), suggesting that the propensity to metastasize and perhaps the capacity of cancer cells to disseminate are more evident at early stages [67]. In support of this evidence, a breast cancer mouse model revealed that small lesions present with a higher dissemination rate to the bone marrow than large primary tumors [10]. This observation was attributed to the gradual increase in the expression of the oncogene HER2, which decreases the progesterone receptor (PR)-dependent early spread signature, favoring tumor growth over dissemination.

Genetic profile comparisons between primary tumors, metastases, and DCCs also support early dissemination. A study involving 743 patients showed that the number of cytokeratin positive (CK+) cells in the bone marrow of patients with early-stage breast cancer was higher than that of control patients with benign breast lesions. In these patients, the presence of CK + cells in the bone marrow was associated with overt metastatic disease and death but not locoregional relapse [32, 68], suggesting different mechanisms for controlling distant and locoregional recurrence. Notably, chromosomal abnormalities were profiled in DCCs from patients at M0 stage, primary tumors, and metastasis, and DCCs exhibited fewer aberrations than primary tumors and metastasis [13]. Similar results were obtained by Schmidt-Kittler et al., who showed that DCCs found in the bone marrow of patients with breast cancer (M0 stage) had fewer genetic alterations than primary cancer cells [34], demonstrating that DCCs seed the bone marrow early in disease progression. Phylogenetic analysis of genomic data comparing normal tissue to primary tumors and metastases for 40 patients with 13 types of cancer, including lung, pancreas, breast, head and neck, and colon cancers, was able to define a tumor evolutionary clock that revealed that metastatic lineages arose early in tumor development [23]. In agreement, dissemination of pancreatic cancer cells from early lesions has been described in a Pdx1-Cre-dependent knock-in mouse model for Kras^G12D^ with a conditional null allele of P53, in presence of a YFP as reporter (also known as KPYC model for Kras^G12D^; p53^fl/+^; Rosa^YFP^; Pdx1-Cre) ([38] and see Table 1 in “Experimental animal models to study early metastatic dissemination” section for more details of the model). In here, eDCCs and early circulating clones have been identified in liver and in blood, respectively.

Similar conclusions were drawn by Hu et al. after profiling the evolutionary dynamics of 118 biopsies from 23 patients diagnosed with colorectal cancer with metastasis to the liver or brain. Using a spatial computational model of tumor growth and metastasis that could determine the age of the primary tumor at the time of metastatic dissemination, they showed driver mutations were acquired early, and in 81% of the cases, early DCCs seeded metastases while the primary tumor was still clinically undetected [21]. Although the phylogenetic tree analyses in this study were not constructed using genomic data from single DCCs but rather overt metastasis, the evolutionary pattern and genetic divergence suggest that the identified clones with proven metastatic capacity arose early in the progression of tumor disease. A follow-up study that included patients with colorectal, breast, and lung cancers estimated that metastatic dissemination occurs 2–4 years before the diagnosis of the primary tumors [22]. Lastly, clonal phylogenetic analysis using breast primary tumors and matched lung metastases from MMTV-HER2 females showed that ~ 80% of metastases derived from eDCCs ([10] and see Table 1 in “Experimental animal models to study early metastatic dissemination” section for more details of the model).

Overall, the above-mentioned clinical and experimental literature supports early dissemination events in a variety of cancers and the potential contribution of eDCCs to the metastatic process.

### Other models of dissemination in advanced tumors

Other models of dissemination have been proposed using phylogenic analyses. Phylogenetic analysis of paired primary tumors and metastases in patients with established esophageal adenocarcinomas revealed that the spread of malignant clones occurs rapidly after emergence in an evolved primary tumor (diaspora theory) [69], suggesting that certain genetic alterations confer these malignant clones with the ability to rapidly disseminate from the primary site. It would be interesting to determine whether specific genetic alterations occurring during the clinically undetectable early stages of esophageal adenocarcinoma and other diseases facilitate early spread. Along these lines, Hafner et al., were able to show that benign human epidermal tumors acquired the same oncogenic mutations seen in malignant melanomas and independent benign lesions from the same patient seemed to share a clonal origin [70]. Similarly, approximately 70% of analyzed DCIS-invasive breast carcinoma pairs shared the same genetic alterations, with trp53 mutation being one of the most common [71]. Interestingly, a specific somatic trp53 mutation (R245W) facilitated breast cancer formation and early metastatic dissemination in a mouse model, arguing that specific mutations can drive a parallel evolution model of metastasis formation [72].

The Big Bang model of tumor evolution proposed in a colorectal cancer model suggests that intratumor heterogeneity arises at the early stages in the absence of clonal selection by a single expansion of fit subclones which may carry metastatic potential [21, 73]. Interestingly, in the above-mentioned Big Bang model, the early subclones were not dominant at the primary tumor [73]. Likewise, in a renal carcinoma model, subpopulations of cells that were not dominant at the primary tumor spread and subsequently became the main contributors to later relapses [74]. However, in this scenario, epigenetic changes were responsible for cellular competition between subpopulations of cells in the primary sites, which led to the displacement of less-fit clones into the circulation which eventually became responsible for subsequent distant relapses. Further studies may shed light on whether the above-mentioned models of dissemination seen in advanced tumors are possibly even at early stages of tumor progression.

## Defining the metastatic potential of disseminated cancer cells

One of the unanswered questions regarding the parallel progression model of tumors and metastasis formulated by Christoph Klein in 2009 [9] pertains to why the size of the primary tumor informs on survival prognosis (e.g. patients with smaller tumors have better survival), considering that the dissemination of future metastatic seeds happens prior to the clinically detectable tumor mass. Perhaps, as proposed by Klein, cumulative signals released from the primary tumor are necessary to favor secondary tumor growth. We would like to propose a complementary hypothesis: in cancers where eDCCs and lDCCs co-exist at distant organs and metastases are founded by eDCCs, we propose that lDCCs activate the latent potential of eDCCs to initiate metastasis. In this scenario, a large primary tumor will spend more time releasing waves of late DCCs than a small tumor, increasing the opportunities for eDCCs reawakening. Thus, the size of the primary tumor could affect relapses, even if eDCCs are the founders of metastasis.

**Fig. 2:**
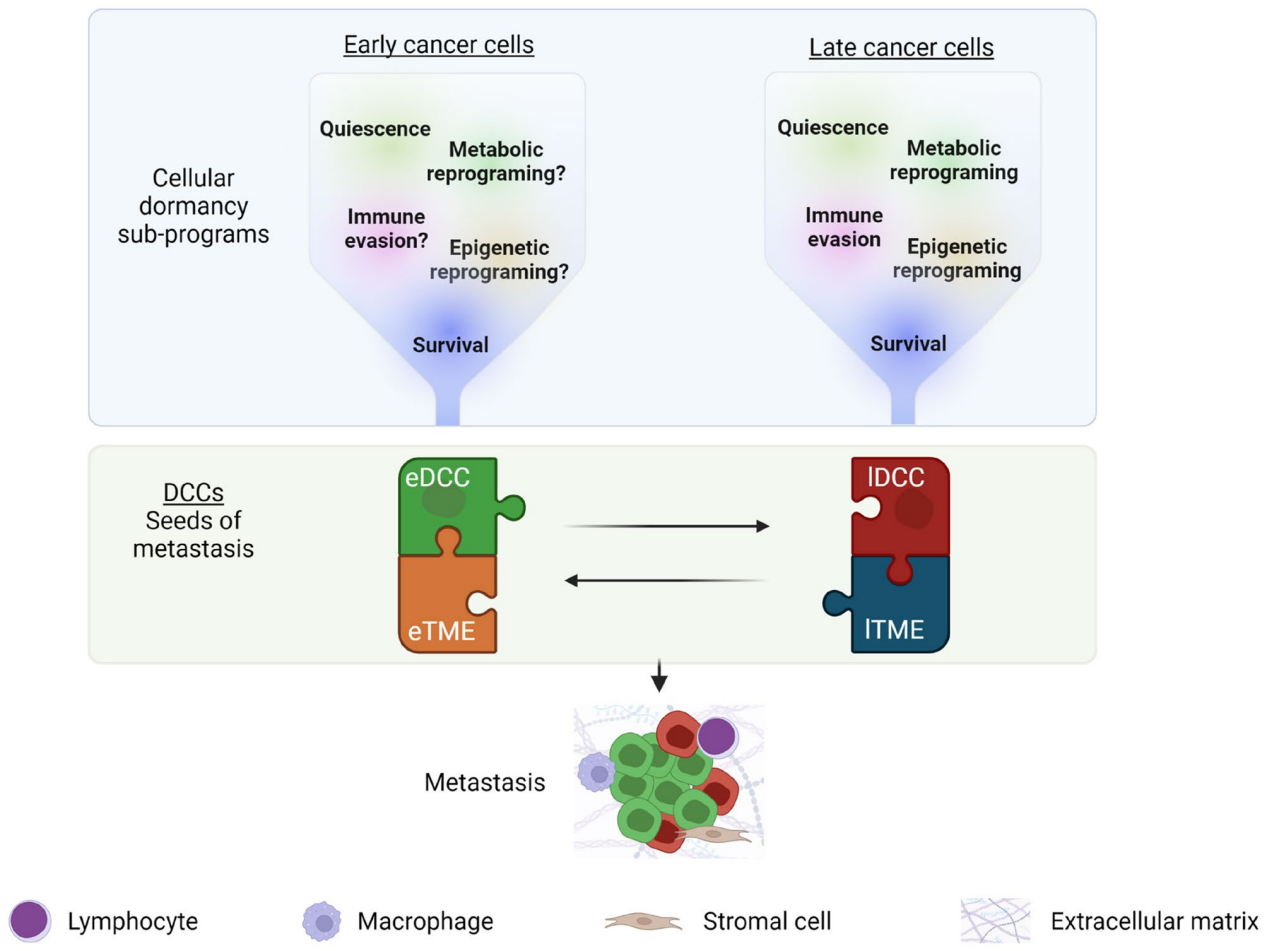
Metastatic potential of dormant DCCs. DCCs that enter cellular dormancy at secondary sites activate different cellular sub-programs, including quiescence, survival, immune evasion, and reprograming at the metabolic and epigenetic levels (see BOX 1 for more detail). These sub-programs have been predominantly studied in late DCCs (due to the accessibility of late models) and are expected to be similarly necessary in eDCCs released from early stages of the disease. These sub-programs create multiple bottlenecks that aid in the selection of cancer cells with potential metastatic capacity. In some types of cancer, the reactivation of eDCCs will be favored. In this context, the presence of eDCCs (green) and, later on, the arrival of late DCCs (lDCCs) might play critical roles in reprogramming their specific tumor microenvironments (TME; eTME for early DCCs and lTME for late DCCs), including immune and stromal cells and non-cellular components such as the ECM. Eventually, the right combination of eDCCs, lDCCs and TME changes will mediate eDCCs reactivation and formation of clinically detectable metastatic disease. (Color figure online)

In cancers where eDCCs found metastases, we propose a new perspective on the definition of the metastatic potential of cancer cells as a temporal crosstalk between distinct evolutionary waves of dormant DCCs and their interactions with the niche (Fig. 2). In this context, similar to the pieces of a puzzle, only the precise combination of genetically less-advanced cancer cells (eDCCs) with genetically advanced tumor cells (lDCCs) and the remodeled microenvironment at the right time will induce the exit from dormancy and metastatic outgrowth of eDCCs (Fig. 2). Therefore, studies investigating the mechanisms of interaction/cooperation between eDCCs and lDCCs may shed light on the biology underlying metastasis formation. Further, research efforts to profile eDCCs and late DCCs, and identify patients carrying these distinct subpopulations of DCCs are of utmost importance for effective therapeutic designs.

## Clinical relevance of minimal residual disease

### Detection of DCCs and current treatment paradigms for patients carrying DCCs

Detecting single DCC or small clusters in patients with cancer is challenging. Standard pathological techniques for the identification of invasion signs and/or the presence of metastasis in draining lymph nodes often fail to detect single DCCs or small clusters of cancer cells. Serial sectioning in combination with immunohistochemistry and immunofluorescence has improved the detection of these events in different types of cancer including melanoma and head and neck squamous cell carcinoma (HNSCC) [8, 37, 75]. Detection of eDCCs in lymph nodes has been shown in patients with early stage lesions. For instance, immunostaining using antibodies recognizing pan-cytokeratin (including cytokeratin 8, 18, and/or 19 by monoclonal antibodies A45-B/B3 or AE1/AE3) [18, 32, 76] has revealed the presence of single or small clusters of DCCs in sentinel lymph nodes in 1–13% of patients diagnosed with DCIS [14, 15, 77–80], supporting early dissemination events. Similarly, Werner-Klein et al., showed detection of eDCCs in sentinel lymph nodes of melanoma patients using anti-gp100 staining [8]. The presence of lymph nodes DCCs has been correlated with increased risk for death [37]. Excision of lymph nodes is not always recommended if draining lymph nodes present a normal appearance under noninvasive imaging techniques in early-stage disease [81]. While it is understandable that no clinician wants to subject patients to procedures that do not offer a high benefit/risk ratio [81], the fact that detection of single cancer cells in metastasis-free organs is linked to poor prognosis in cancers such as melanoma [37] raises the question of whether excision of lymph nodes with a normal appearance should be recommended to better assess long-term disease progression.

Similarly, multiple clinical studies have shown that detection of DCCs in the bone marrow has been associated with metastatic relapse in patients diagnosed with cancers of the lungs [39], prostate [82], colon [83], pancreatic [84], esophageal [85], and breast [86]. When performed, and as in the lymph nodes, detection of DCCs in the bone marrow relies on immunostaining using antibodies against markers of epithelial lineage such as cytokeratins (including cytokeratin 7, 8, 18, and/or 19 by antibodies A45-B/B3, AE1/AE3 or 2E11) or sialomucin (E29). Importantly, bone marrow DCCs have been detected in 14–31% of DCIS patients supporting events of early dissemination from DCIS stages [13–18]. A large clinical study including 10,307 patients with breast cancer (ranging from T1-4, N1-3, M0) recruited from 10 different centers across Europe and the United States was able to confirm what previous and smaller studies had suggested [87–98]: DCC detection in the bone marrow is an independent prognostic marker for overall survival (OS), disease-free survival (DFS), and distal disease-free survival (DDFS) [86]. Despite methodological differences across participating centers (antibody choice and sample preparation) that might have affected detection rates (range from 13 to 48%, with 27% overall detection rate), a total of 2814 patients were DCC positive. With a median follow-up of almost 8 years, the authors concluded that patients with DCC detection in the bone marrow had lower OS and DDFS even at early stages of disease (T1 and N0) and there was no association with locoregional relapse-free survival. Interestingly, the DFS value of DCCs in the bone marrow was lost when patients were treated with neoadjuvant therapy targeting HER2, suggesting HER2 targeting might be controlling DCC capacity to form macrometastasis. Overall, this report suggests that bone marrow aspiration could be collected at time of primary tumor resection, with minimal discomfort to patients while under anesthesia, and be informative to determine which patients need a closer follow-up. In addition, and since a significant fraction of patients with overt tumors and DCC detection did not progress to metastatic disease during the follow up period, it can be speculated that the nature of dormant DCCs at secondary organs is indeed heterogenous. Hence, identifying which cancer cell population is relevant for disease progression will offer a clearer vision for the design of targeted therapies.

Simultaneously with the emergency of new mechanisms describing dissemination, dormancy, and reactivation of DCCs in pre-clinical models, new clinical strategies are being developed to target DCCs, increase survival, and reduce the risk of death in patients with cancer. Bisphosphonates have been extensively studied and were originally reported to prevent bone damage by inhibiting osteoclast-mediated resorption. Zoledronic acid (ZOL), one of the most potent bisphosphonates, has been shown to improve DFS in postmenopausal patients with breast cancer when administered immediately after or following a delay after letrozole administration (ZO-FAST phase III study involving 1065 metastasis-free patients at stages I–III with ER+/PR+breast cancer) [99]. ZOL-treated patients also developed fewer local and distal recurrences, suggesting that DCCs or their microenvironments may be targeted by ZOL. Following this, Arf et al. showed that the proportion of patients with DCC detection after combined chemotherapy and ZOL administration was less than that in patients treated with chemotherapy alone (clinical breast cancer stages II–III; phase 2 randomized study; N = 120). However, this difference was not statistically significant [100]. In another study, Vidula et al. [101] showed that when patients with breast cancer at stages I–III were treated with ZOL after adjuvant therapy (chemotherapy and tamoxifen or an aromatase inhibitor when classified as hormone receptor-positive), a significant reduction in DCCs in the bone marrow at 12 and 24 months post-treatment was observed. Although this study included only 45 patients and did not have a control arm, the authors also reported a DCC clearance rate of approximately 30%. A different study showed that ZOL increased DCC clearance in patients with early breast cancer (pT1-4, N1-2, M0) after 12 months of treatment with a combination of adjuvant therapy and ZOL when compared to the control arm receiving adjuvant therapy alone (N = 96) [102]. Lastly, Banys et al. demonstrated that a significantly smaller number of patients that received ZOL treatment and adjuvant therapy compared to adjuvant therapy alone developed metastatic or recurrent disease during follow-up [103] (NCT00172068). Whether these strategies can target the bulk or specific subsets of DCCs in bone marrow or other secondary organs still needs to be investigated. Moreover, their mechanisms of action remain to be studied; however, some evidence proposes that they could inhibit osteoclastic bone resorption, which reactivates dormant DCCs [104].

Similar to ZOL, denosumab, a monoclonal antibody targeting RANKL, a protein that mediates bone resorption, was evaluated in combination with neoadjuvant or adjuvant chemotherapy to determine whether disease outcomes in women with high-risk early breast cancer improved (Phase 3 D-CARE trial, stages II–III, no DCC status evaluated). No improvement was observed [105]. Researchers have also proposed evaluating whether denosumab has an effect on DFS through DCC targeting/depletion when administered after completion of adjuvant or neoadjuvant therapy, in a strategy parallel to that reported by Vidula et al. [101]. However, the trial was terminated because of low accrual (NCT01545648). Other strategies to target minimal residual disease (MRD) have also been proposed but have not yet reported their findings or failed to obtain funding or patient recruitment. These include the evaluation of the efficacy of trastuzumab for the elimination of HER2+DCCs (NCT01779050; terminated because of loss of funding); the use of gedatolisib (PI3Kα/γ and mTOR inhibitor), abemaciclib (CDK4/6 inhibitor), and hydroxychloroquine (autophagy inhibitor) to target DCCs in the bone marrow (NCT04523857; no results published yet); and even the mobilization of DCCs out of the bone marrow in prostate cancer by treatment with burixafor hydrobromide (CXCR4 inhibitor), which would render them more susceptible to chemotherapy (NCT02478125; terminated because of low accrual). In 2021, a new phase 2 clinical trial was initiated to target bone marrow DCCs using hydroxychloroquine (autophagy inhibitor) or avelumab (PD-1/PD-L1 blocker), with or without palbociclib (CDK4/6 inhibitor), in patients with early-stage ER + breast cancer (NCT04841148). This clinical trial is ongoing.

Another strategy aims to maintain the dormant state of DCCs. In this case, a phase II trial evaluated whether treatment with a combination of 5-azacitidine (5-AZA) and all-trans-retinoic acid (ATRA), FDA-approved drugs for the treatment of myelodysplastic syndrome [106] and acute promyelocytic leukemia [107], respectively, could inactivate prostate cancer cells and maintain the disease in a dormant state. For this purpose, patients with prostate cancer who presented biochemical recurrence (no evidence of cancer on radiographic scans but with rising prostate-specific antigen (PSA) levels) were recruited and treated with 5-AZA and ATRA. To date, no results have been published (NCT03572387).

Finally, DCC detection could be used as a decision-making tool when monitoring disease response to treatment and identifying biomarkers for outcome determination. In this regard, Naume et al. and others identified high-risk patients with early breast cancer and persistent DCCs after the first round of treatment and applied secondary treatments to eliminate all DCCs, emphasizing their potential as surrogate markers for monitoring treatment efficacy [31, 108]. It is notable that although the presence of DCC predicted recurrence in patients with breast cancer over the following five years [68], 60% of DCC-positive patients remained free of disease during that period. Thus, the identification of biomarkers that can describe the progression of MRD is urgently needed. To this end, Borgen et al. stained bone marrow aspirates from 86 DCC-positive patients for NR2F1, a dormancy inducer, and the Ki67 proliferation marker [109]. The findings revealed that NR2F1 serves as a biomarker for predicting long-term recurrence (within five years), whereas Ki67 staining does not. These findings increase the necessity to perform dormancy marker profiling in MRD. Not only cell intrinsic factors in DCCs may explain why some DCCs take longer to reactivate but also environmental signals could determine DCC fate, such as the presence of inhospitable microenvironments or antimetastatic niches [110]. A clear example of an inhospitable microenvironment for DCCs is the skeletal muscle. Different mechanisms can explain why muscle is arguably the most resistant tissue to metastasis. These include the biomechanical destruction of cancer cells [111], the presence of soluble factors such as adenosine receptor agonists [112], the lineage specification of cancer cells [113], and oxidative stress [114] in skeletal muscle microenvironments. Some of these mechanisms may also explain the low metastatic burden in other organs, such as the spleen, thyroid, and yellow bone marrow [110]. Understanding the factors that make the environment conducive as well as the plasticity of DCCs to adapt to such conditions will open new opportunities for targeting DCCs before they exit dormancy and establish overt metastatic tumors. Further, if we assume that eDCCs derived from early stages of tumor progression have fewer genetic changes, it may be possible that strategies based on the underlying biology of genetically advanced invasive tumors may neglect eDCCs; thus, new therapeutic strategies to eradicate eDCCs are needed. To achieve this, we first need to characterize these patient-derived eDCCs and later be able to identify patients carrying eDCCs before they develop metastasis. These characterization efforts, which have recently started in animal models [12], will translate into the identification of the specific biomarkers that we urgently need to stratify patients at risk and to potentially, develop new therapies.

### Detection of circulating cancer cells in early disease

One of the intermediate steps of the metastatic cascade involves the ability of tumor cells to enter and survive in the circulation as circulating cancer cells (CCCs) and extravasate into the surrounding tissues. It is reasonable to assume that CCCs detected in patients diagnosed with primary tumors and metastases are derived from both the primary tumor and metastases, and if surgery has been performed on the primary tumor, then CCCs are derived from metastases. During remission, the detection of CCCs may represent a clinically undetectable recurrence [115]. Interestingly, CCCs have also been detected in patients with benign and early disease, such as pancreatic cystic lesions [116], intestinal polyps [117–119], chronic lung diseases [120], and DCIS [121]. However, the metastatic potential of these early CCCs is largely unknown.

Pancreatic ductal adenocarcinoma (PDAC) is associated with high mortality and a poor prognosis [122]. It is generally asymptomatic in its early stages, limiting the efficacy of current therapies. Furthermore, the diagnosis of early disease is rare in the general population and relies heavily on imaging techniques used during the evaluation of unrelated conditions [123]. Thus, the use of CCC detection has been proposed as a tool for screening and early detection of PDAC. Using GEDI (Geometrically Enhanced Differential Immunocapture; a microfluidic device in conjunction with DAPI, CD45, CK19, and Pdx-1 staining) [124], circulating pancreatic cells were detected in 33% of patients diagnosed as cancer-free; however, these patients had intraductal papillary mucinous neoplasm (IPMN), a type of cystic lesion [116] considered a precursor of PDAC [125]. Later, independent groups identified circulating pancreatic cells in patients that were undergoing surgical resection of precursor cystic lesions (mainly IPMN) in up to 88% of cases evaluated [126–128] using previously validated CCC detection systems, including ScreenCell® [129], ISET© [130], and CTC-iChip [131]. Interestingly, similar detection rates of CCCs were observed in benign, premalignant, and malignant lesions, suggesting that early evolved cells efficiently disseminated from the primary site [126]. Whether CCCs from benign and/or premalignant pancreatic lesions can be used as a risk factor for future distant relapses remains to be determined.

Using previously validated CCC detection systems, including EPISPOT™ [132] (CK19 +in CD45-depleted cells) and CellSearch [133] (CK+after epithelial cell adhesion molecule (EpCAM) enrichment), positive events that met the criteria for “cancer cells” were detected in a study of 53 patients diagnosed with benign colon diseases (diverticulosis, benign polyps, and Crohn’s disease, among others). Although the metastatic potential of these cells or tumor development in these patients did not occur within three years of follow-up [118], this does not rule these cells out of possessing seeding and/or metastatic potential. In fact, these benign diseases are a risk factor for the development of colorectal cancer [134]. An independent study detected circulating epithelial cells in 33% of preoperative patients with colorectal polyps (compared to only 8% shown in a previous study [118]) by using a more sensitive CMx platform (EpCAM-based capture system and subsequent corroboration by staining against CK20) [119]. Thus, intestinal cells from the earliest type of benign lesion are also capable of dissemination, and whether they can predict the risk of metastatic disease needs to be evaluated.

Although challenging, some clinical trials have shown not only the presence of CCCs in early disease but also their prognostic value for developing tumors. For instance, patients diagnosed with chronic obstructive pulmonary disease (COPD), a risk factor for lung cancer, were monitored for the presence of circulating cells with malignant cytopathologic features. In 3% (5 of 168) of the patients, CCCs were identified and isolated using ISET technology, corroborated by May Grunwald Giemsa cytological analysis and immunostaining for pan-cytokeratin and vimentin. Patients with detectable CCCs did not present lung nodules at the time of COPD diagnosis. However, these were detected at follow-up visits, and all five patients developed tumors within 1–4 years (80% invasive carcinomas and 20% squamous cell carcinoma), demonstrating the predictive value of CCCs in early non-small cell lung carcinoma (NSCLC) [120]. Since NSCLC presents metastatic lineages arising early in tumor development [23], one could ask whether the CCCs detected during COPD could seed future relapses.

## Mechanisms of cancer cell dissemination

### Partial EMT is linked to dissemination and metastatic colonization

It has been proposed that epithelial-mesenchymal transition (EMT) plays an essential role in metastatic dissemination. The expansion of our understanding of EMT markers and transcriptional profiling led us to arrive at the idea that EMT is not dichotomous but rather a spectrum of phenotypes between epithelial and mesenchymal states that cancer cells exploit for their survival, migration, metastatic capacity, and even resistance to therapy. Although the definition, mechanisms governing these hybrid states, and their relevance have not been explored in detail, some studies have begun to show the association between hybrid states and poorer prognosis [135, 136] and enhanced metastatic capacity [136–138].

Transcriptomic analysis of 7180 primary tumors of epithelial origin spanning 25 cancer types from the harmonized version of TCGA using the TCGAbiolinks R package (pan-cancer analysis) reconstructed EMT pseudotime trajectories that suggested three macro-states: epithelial, hybrid, and mesenchymal [139]. The hybrid state was surprisingly frequent among the samples (39%), stable over time, associated with aneuploidy, which is correlated with a poor prognosis [140, 141], and associated with a worse overall survival outcome. Although it is probable that this hybrid state exists within a spectrum of possible states, its stability suggests that this state is energetically favorable, and because increased evidence suggests that hybrid EMT is associated with stemness, chemoresistance, immune evasion, and metastasis, this hybrid state may be relevant in cancer disease [135, 136, 142, 143].

Numerous studies focus on the protein E-cadherin, an adhesion protein in the calcium adhesion superfamily that is mainly expressed in epithelial cells and maintains cell adhesion and epithelial structural integrity. Its loss is a key marker of EMT, which has been linked to an increased risk for metastasis [144–146]. However, emerging data on the E-cadherin functions suggests a context-dependent role (i.e. different stages of metastasis, different cancer types) and its downregulation does not always imply upregulation of all well-known EMT mesenchymal traits but perhaps the development of hybrid EMT states. For instance, downregulation of E-cadherin has been linked with a lower metastatic burden and the induction of dormancy. In this regard, Aouad et al. recently demonstrated that E-cadherin reduction in an ER + breast cancer model (MCF7 cells) induced slow proliferation and apoptosis at the primary site and reduced the lung metastatic burden via dormancy induction [147]. Interestingly, ectopic expression of E-cadherin in dormant DCCs was sufficient to drive DCCs out of dormancy. In this study, the reduction of E-cadherin increased zinc finger E-box-binding homeobox 1 (ZEB1) transcripts, whereas the expression of other EMT markers was unaffected. An elegant study using the luminal invasive ductal carcinoma MMTV-PyMT mouse model demonstrated that loss of E-cadherin inhibited metastatic burden [148]. Transcriptional analysis using RNAseq in E-Cadherin + vs. E-cadherin- cells showed no significant changes in the expression of canonical EMT transcripts, suggesting that E-cadherin function does not require a complete loss of epithelial or a complete gain of mesenchymal traits in this model. Similarly, pancreatic dormant DCCs found in human and mouse liver sections became negative for E-cadherin without upregulating EMT markers (Desmin, alpha smooth muscle actin (aSMA), Snail1, or Slug) [53].

Additional studies provide evidence of the enhanced metastatic capacity of hybrid EMT states. For instance, Lui et al. showed that ex vivo cultured CCCs with a hybrid phenotype had a higher metastatic capacity than epithelial or mesenchymal-type CCCs [143]. Interestingly, different grades of hybrid phenotype were identified, with CCCs that were more epithelial than mesenchymal type (higher E-cadherin and EpCAM protein levels) having higher metastatic potential. Using an ER-/PR- breast cancer cell line, Brown et al. [136] isolated three hybrid EMT clones and demonstrated that these intermediate clones had higher tumor-initiating capacity than fully mesenchymal phenotypes. Notably, the hybrid and mesenchymal EMT clones had a latency in growth when compared to parental cells, and this lag was more evident in mesenchymal clones with very low E-cadherin levels. Moreover, Pastushenko et al. [138] showed that hybrid EMT subpopulations (with higher E-cadherin messenger levels) derived from skin tumors had higher metastatic potential than more mesenchymal subpopulations (with lower E-cadherin messenger levels). In line with these results, Cui et al. recently showed that loss of MLL3, a histone methyltransferase, increases metastatic colonization and enriches metastasis with hybrid Vimentin+/E-cadherin + cells when compared to MLL3 wild-type mesenchymal (Vimentin+/E-cadherin-) breast cancer cells [137]. Thus, one hypothesis could be that mesenchymal and hybrid EMT DCCs with low levels of E-cadherin may represent clones more susceptible to a longer dormancy phase, whereas hybrid EMT clones with high E-cadherin levels may immediately (or after a short dormancy period) form metastases. This may not be the case in PanIN lesions where E-cadherin-negative PanIN cells injected into the pancreata of NOD/SCID mice formed tumors faster (2 months post-implantation) than E-cadherin-positive cells (4 months) [38]. Further, PDAC metastatic clones having a more mesenchymal-like hybrid phenotype (lower E-cadherin levels) were more metastatic and disseminated more than epithelial-like hybrid-EMT (higher E-cadherin levels) [149]. Worth mentioning is that PDAC hybrid EMT clones were still more metastatic than the EMT extremes.

Our work in early premalignant lesions from the MMTV-HER2 animal model (see description of the model below and Table 1) described a partial/hybrid EMT at the primary site, downstream of HER2, that mediates in vivo dissemination to the lungs (determined by intravital imaging and quantification of DCCs in the lung at the single cell stage 1–2 weeks post orthotopic injection) [150]. We showed that HER2 downregulated the expression of the NR2F1 nuclear receptor via p38, and loss of NR2F1 triggered a partial/hybrid EMT, characterized by the maintenance of luminal epithelial markers, such as cytokeratin 18, a limited suppression of *Gata3* mRNA expression, and downregulation of E-cadherin, while expression of CK14, TWIST, and PRRX1 was induced. No changes were observed in other EMT markers such as VIMENTIN, SNAIL1, and AXIN2. Once in the lungs, these HER2 + eDCCs underwent a period of dormancy (approximately 90% Ki67 negative) [11, 12], and they were enriched in mesenchymal- and hybrid-like EMT signatures, with low E-cadherin mRNA levels [12], nearly undetectable by immunofluorescence (IF) staining [11] (Fig. 1). Interestingly, these HER2 + eDCCs eventually reactivated and were responsible for ~ 80% of lung metastasis [10]. Based on the above-mentioned studies on breast cancer, we can postulate that hybrid dormant HER2 + eDCCs are more sensitive to reactivation signals than the mesenchymal-like type and, therefore, are the first type of eDCCs to transition back to the epithelial phenotype (acquiring high E-cadherin levels), exit dormancy, start proliferating, and form metastases. Undoubtedly, this hypothesis needs to be validated using in vitro models as well as in vivo models such as the ones described below and in Table 1.

Overall, the above studies suggest that in certain types of tumors, cancer cells with hybrid EMT may be clinically more relevant than full mesenchymal states and may rapidly develop into deadly metastases. Further investigations are required to determine the role of hybrid EMT states in all types of dormant DCCs.

## Experimental animal models to study early metastatic dissemination

In vitro studies have been essential to address some of the biology of the early dissemination process. For example, 3D Matrigel cultures allows to address questions on motility, invasion, EMT, and co-cultures as we shown in [10–12, 150]; scaffolds that mimic different microenvironments are used to explore the behavior of single eDCCs [151–154]; microfluidics devices to mimic intra- and extravasation processes could also address fundamental questions on eDCCs behavior [155, 156]; and computational models can model the process of metastasis (including tumor cell-bloodstream dynamics) and interactions of tumor cell clones with the secondary microenvironment [157]. However, these methods isolate different aspects of a multi-step process that is difficult to integrate in the absence of in vivo models. Few mouse models have been described for the study of early dissemination and eDCC biology. In the following section, we aim to summarize the details of the transgenic models, the time of cancer cell dissemination, the sites of early dissemination, and the potential role of the immune system in dissemination and dormancy (Table 1). Ultimately, the animal model provides the opportunity to take into account all and every single step of the metastatic process (dissemination, EMT features, survival at new niches, dormancy state, reactivation and metastasis formation) and in a way that closely resembles the spontaneous biology of DCCs.

### MMTV-HER2 breast cancer model

Dissemination from early cancer lesions in the absence of invasive histopathology features has been more commonly studied in MMTV-HER2 animal models. These animals develop spontaneous mammary tumors and metastases after overexpression of HER2 (neu/ErbB2) in the mammary gland epithelia, making them a suitable model to study HER2-driven tumorigenesis. The first transgenic mice were developed in the Swiss-Webster background by Muller and colleagues in 1988 [158]. The MMTV/c-neu mouse model carries an activated rat HER2 oncogene with a V664E point mutation in the transmembrane domain that favors the aggregation and activation of the receptor in the absence of a ligand [159–161]. In the BALB/c background, activated HER2 is first detected at the onset of puberty (3–4 weeks of age). Hyperplastic lesions are found around 7–9 weeks of age [162], while palpable in situ carcinomas are detected between 14 and 18 weeks of age. Five to ten weeks later, the tumors become invasive, and metastatic foci are detectable macroscopically [13]. In this model, hyperplastic cells express ER/PR, which are later lost at advanced stages of tumor progression [10]. In 1992, the same group generated a second model in the FVB background carrying the wild-type HER2 allele under the MMTV promoter [163]. These animals are also characterized by the development of focal mammary tumors, albeit with a longer latency than in the activated HER2 model. Furthermore, these animals develop spontaneous mammary tumors around 30 weeks of age [163], which progress from hyperplastic lesions that arise around 16–18 weeks of age [11, 12]. Both models offer an accessible time window for the study of early dissemination and therefore, the biology of DCCs in dormant and reactivated states.

In both models (wild-type and activated HER2), at an early age, a subpopulation of HER2 + early cancer cells characterized by low levels of phospho-p38 and its targets p-ATF2, p-MK2, and p-Hsp27, low levels of E-cadherin, high levels of Twist, and a progesterone (PR) signature showed enhanced invasive activity and dissemination capacity when compared to normal mammary epithelium [10, 11, 164]. These early cancer cells activated a program that triggered a partial EMT transition (without complete loss of the epithelial phenotype) that was dependent on HER2, Wnt, and PR signaling [10, 11]. A recent study revealed that the orphan nuclear receptor NR2F1 was negatively regulated by HER2 and positively regulated by p38alpha, and its expression prevented the invasion and dissemination of MMTV-HER2 early cancer cells [150].

The groups of Christoph Klein and Julio Aguirre-Ghiso have shown that HER2 + early cancer cells have metastatic potential [10, 11, 13]. This was achieved by performing several experiments that measured lung metastasis, such as: (a) surgery of the entire milk line after detecting in situ carcinoma in transgenic females [13]; (b) in wild-type females that received temporary orthotopic grafts of mammary glands from donors with in situ carcinoma [13]; (c) injection of early cancer cells into the mammary fat pad of syngeneic and/or nude mice [10–12]; and (d) injection of early cancer cells into the tail vein of animals [11, 12].

In the HER2 mouse models, eDCCs can be detected by HER2 + staining in the lungs and bone marrow soon after the activation of HER2 during stages of hyperplastic lesions [10, 11, 13]. Single-cell comparative genomic hybridization of the bone marrow eDCCs showed chromosomal aberrations which traced them back to the early hyperplastic lesion [13]. Once in the lungs, eDCCs remained dormant (~ 90% Ki67 negative) for approximately 15–18 weeks and exhibited a hybrid and mesenchymal EMT phenotype [11, 12]. Importantly, genetic profiling using comparative genome hybridization of primary tumors against matched metastases suggested that up to 80% of the metastatic burden in these MMTV-HER2 animals was derived from eDCCs [10]. This result suggests that eDCCs are the main contributors to lung metastases in this model.

Early dissemination events in the MMTV-HER2 animal model also required the participation of macrophages, as their depletion, mediated by CSF1R blockage only during the hyperplastic lesion stage, reduced dissemination, and metastatic burden [33]. Macrophages, normally found in the stroma around the mammary ducts, entered the ductal epithelial layer in early lesions when the epithelium was characterized by hyperplasia and mammary intraepithelial neoplasia [33]. This effect was mediated by CCL2, a potent macrophage chemoattractant produced by HER2 + early cancer cells, among other cells. In response to CCL2 signaling, the presence of macrophages disrupted E-cadherin expression in the surrounding epithelial cells through macrophage-dependent Wnt1 signaling. Furthermore, HER2 + early cancer cells efficiently formed *tumor microenvironment of metastasis* (TMEM) structures that have been shown to serve as portals for intravasation and have clinical prognostic value in human disease [44, 165]. The detection of intra-lesion macrophages was confirmed in a small cohort of patients with DCIS, and this had no correlation with HER2 status [33].

In summary, these studies have demonstrated HER2 + eDCCs as a new source of metastasis.

### MMTV-PyMT breast cancer model

Early dissemination has also been described in an MMTV-PyMT animal model. This animal model is characterized by the expression of the polyoma virus middle T antigen (PyMT) under the mammary tumor virus LTR and is therefore restricted to the mammary gland [45]. Rapid multifocal mammary adenocarcinomas spontaneously develop in these animals and are commonly detected at approximately 14 weeks of age. Hyperplastic early lesions can be detected as early as 4 weeks of age [46]. Although PyMT is not expressed in humans, its oncogenic capacity activates a range of signaling pathways, including Src, Ras, and c-Myc, which are commonly altered in human diseases. In this model, tumors arise from the luminal cells of the mammary gland and resemble human disease as they lose ER and PR, overexpress ErbB2 [46], and exhibit similarity to luminal B tumors according to genetic profiling [47]. Remarkably, despite their rapid progression, eDCCs can be detected by colony formation assays from digested lungs as early as 4 weeks of age, when the histological evaluation of the mammary tree appears normal [48]. Unequivocal identification of eDCCs was demonstrated by Christoph Klein in 2008 by IHC using a GP11 antibody recognizing both CK8 and CK18 staining in bone marrow cytospins and by histology in lung sections [13]. These eDCCs are associated with stem cell markers, which could explain their tumorigenic capacity [48].

### Mouse intraductal (MIND) DCIS xenograft models

The intraductal human-in-mouse transplantation model allows the in vivo study of DCIS malignancy. Human DCIS cell lines, primary DCIS, and atypical hyperplasia cultures derived from patient’s biopsies can be implanted in the mammary ducts of immunocompromised mice [50, 51]. Primary DCIS and atypical hyperplasia cells derived from patient’s biopsies recapitulate, in the MIND xenografts, the expression patterns of human cytokeratins, ER, and HER2, which are specific to human cells, pathology, and the heterogeneity of human DCIS disease. The advantage of MIND xenografts of primary human DCIS is that they do not progress to invasive carcinoma for the first 8 weeks, which offers a time window to specifically study DCIS biology. Potentially, the use of MIND DCIS xenografts might provide insight into which patients will progress to invasive breast cancer and reduce overtreatment of patients who are not at risk of progression. However, because the animals are immunocompromised, early lesions and invasive carcinomas will be developed in an environment that will not fully recapitulate human tumor biology or pharmacodynamics, which needs to be carefully considered. Further, this model is compatible with the use of the human cell line SUM-225, which has been shown to resemble HER2 + DCIS lesions, and the MCF10DCIS.COM line which establish basal-like DCIS lesions. The SUM-225 HER2 + DCIS lesions invade the myoepithelial layer 14 weeks after transplantation and the MCF10DCIS.COM DCIS lesions become invasive after 10 weeks [52]. In both cases, a significant time window for the study of eDCCs is available. The molecular characterization of the subpopulations of cells in DCIS lesions that enter the circulation, reach distant sites, and eventually form metastases (eDCCs responsible for DCIS breast cancer mortality [19]) is of utmost importance for the prevention of metastasis not only in patients with DCIS but also in all types of cancers with early spread.

### KPC mouse model

The dissemination of pancreatic cancer cells from intraepithelial neoplasia has been described in a Pdx1-Cre-dependent knock-in mouse model for Kras^G12D^ with a conditional null allele of P53, in presence of a YFP as reporter (also known as KPYC for Kras^G12D^; p53^fl/+^; Rosa^YFP^; Pdx1-Cre) [38]. The advantage of these animals is that they exhibit histological and molecular resemblances to human disease [38]: they developed pancreatic intraepithelial neoplasia (PanIN) lesions at around 8–10 weeks of age with detection of CCCs in the blood and eDCCs in the liver, before evidence of carcinomas at the primary site. Detection of PDAC (pancreatic ductal adenocarcinoma) occurs at 16 weeks. In this mouse model, inflammation was shown to contribute to the EMT phenotype and early dissemination. The metastatic potential of eDCCs in the KPYC mouse model still needs to be demonstrated, although clinical evidence strongly suggests the relevance of these events: most patients present metastatic disease at time of diagnosis [166] with metastatic lesions having low proliferation rate compared to the primary site [167, 168], but similar sizes at the time of diagnosis, suggestive of parallel evolution and early dissemination. The KPC animal model, carrying knock-in expression of KRAS^G12D^ in combination with constitutive p53 deletion mediated by Cre recombinase (under Pdx1, P48, or Nestin promoters), presents PanIN lesions at a young age (8–10 weeks of age), with rapid development of PDAC (10–30 weeks) that requires monitoring by non-invasive imaging techniques or manual palpation [54]. Metastasis to multiple organs are developed with variable latency.

A slower progressor model is the KC model, a knock-in expression of KRAS (Kras^G12D^) by Cre recombinase under the pancreatic promoter P48, is characterized by rapid development of PanIN lesions in the first weeks of life that resemble human PanINs I–III, with longer latency before PDAC is detected at around 30 weeks of age [55, 61]. The disadvantage of this model is that KRAS mutation alone is rarely found in human disease.

### RET.ADD melanoma model

A spontaneous uveal melanoma mouse model called RET. AAD [62] also exhibits early metastatic dissemination [35]. In this model, an activated human RET oncogene is expressed in melanocytes under the mouse metallothionein (MT)-I promoter/enhancer that triggers melanosis and melanocytic tumors [169], in presence of the chimeric MHC class I molecule AAD. The RET.AAD animal model recapitulates melanoma progression, including metastatic stages. In this model, eDCCs are observed in the lung of 6–7-week-old C57BL/6 mice by immunohistochemistry (IHC) targeting the melanocyte marker S100B and in visceral organs as early as 3 weeks of age when determined by qRT-PCR detection of the dopachrome tautomerase (*Dct*) gene. The dormancy periods between DCC arrival and overt metastasis vary depending on the secondary organ, with metastasis in the lungs becoming evident at 1 year of age on average, offering one of the main advantages of this model for the study of early dissemination and dormancy. Genome wide SNP profiling comparing somatic mutations in primary and metastatic tumors from the same animals suggests that dissemination of tumor cells occurs during the hyperplastic stages [35]. Histopathological analysis of the lesions in the choroids of young animals (2–4 weeks of age) revealed only hyperplastic lesions. Interestingly, small nodules were found in the sclera at 2 weeks of age. Therefore, metastasis latency in the sclera is very brief (~ 2 weeks), but more importantly, cells that locally migrated to the sclera were only derived from hyperplastic lesions found in the choroids. When the RET.ADD model is generated in Balb/c mice there is a more pronounced latency for primary and metastatic disease [63], with DCC presence in secondary organs expected to be delayed as well. Despite it resemblance to human disease, the activated version of the RET oncogene in this animal model is not found in humans, which may limit the translatability of mechanisms and biomarkers of dormant melanoma eDCCs that could be identified in the future. The RET.AAD model also expresses chimeric MHC AAD (alpha1-alpha2 domains of the HLA-A2 linked to the alpha3 domain H2-Dd) that allows the monitoring of CD8 + T cells. The role of CD8 + T cells in maintaining dormancy of eDCCs in visceral and lung metastasis formation has been proposed, as depletion of CD8 + T cells in 6-week-old RET.ADD mice (by administering neutralizing antibodies) led to rapid development of micrometastases [35], arguing that CD8 + T cell depletion may facilitate the exit of eDCCs from dormancy.

### Concluding remarks

Secondary organs might accumulate evolutionarily distinct types of DCCs, starting at the early and asymptomatic stages of tumor progression. These multiple waves of DCCs can remain dormant for decades or even the lifetime of patients (e.g. patients diagnosed with only non-invasive early diseases such as DCIS). Considering a niche where different waves of DCCs co-exist, several questions arise. For example, what type of DCC, early or late (or interactions/cooperation between them) initiate the first escape from dormancy. Further, if eDCCs are the first to reactivate, do they switch to a transcriptomic/genomic profile that resemble late DCCs (more epithelial-like/more genetically evolved)? Regarding the remodeling of the microenvironment, which types of DCCs (early or late) regulate the switch from an evasive immune niche to a pro-tumorigenic niche; which types of DCCs induce a pro-metastatic extracellular matrix remodeling. Exploring the use of in vitro, in vivo, and computational models that can recapitulate the behavior of DCC waves—their migration from their primary sites to distant sites, acquisition of different EMT phenotypes in this process, and communication with new niches and each other to survive, remain dormant, and ultimately reawaken—will aid in the design of new therapies to prevent deadly metastatic disease. It is also essential to factor in the contribution of the tumor microenvironment, including its organ- and patient-based diversity, together with the effect of current standard-of-care treatments on the biology of dormant DCCs. For example, different organs may induce different metabolic fitness in DCCs [170] (BOX 1). Further, targeted therapy may exacerbate metabolic vulnerabilities in DCCs, as shown for loco-regional persistent breast cancer cells [171]. Lastly, eDCCs might manifest distinct responses to treatment compared to genetically advanced DCCs. Because our knowledge of the biology, origins, and heterogeneity of DCCs is still limited for most cancer types, research efforts to profile eDCCs and lDCCs are of utmost importance for identifying patients at risk of relapse, reducing overtreatment and designing effective therapeutic strategies.

BOX 1-cellular dormancy of DCCs Cellular dormancy is defined as the state during the cell life cycle of a DCC when proliferation stops; however, instead of being a passive state (e.g., a responsive mechanism to the lack of mitogenic signaling), increasing evidence suggests that it is an active program that involves the following five sub-programs:

**1. Quiescence**

Once they arrive at secondary sites, DCCs arrest in the G0-G1 phase for extended periods of time [172–174]. This cell cycle arrest is a consequence of intrinsic cellular signals and niche-induced quiescence. Notably, the quiescence sub-program can overwrite the presence of oncogenic mutations, thereby acting as a tumor suppressor [175].

**2. Epigenetic reprogramming**

Similar to embryonic, pluripotent, and cancer stem cells, dormant cancer cells can alternate between periods of dormancy and active proliferation. The capacity of dormant DCCs to resume growth might depend on their potential to halt several pathways that are engaged but not active. Adult and embryonic quiescent stem cells rely on a poised chromatin landscape and post-transcriptional regulation mechanisms (including microRNAs and RNA-binding proteins) to respond rapidly to activating signals [176–182]. However, whether dormant DCCs maintain a latent metastatic capacity via the same mechanisms remains to be explored. Furthermore, dormant cancer cells present higher percentages of repressive chromatin compared to their proliferative counterparts [183]; however, the chromatin landscape seems to be permissive to the transcription of a quiescence signature. Interestingly, spontaneous dormant DCCs upregulated members of the H2A family of histones, e.g., macroH2A variants, and regulated transcriptional quiescence and senescence-like programs while inducing a global repressive chromatin state [184].

**3. Metabolic reprogramming**

Persistent dormant tumor cells can rely on oxidative stress (OXPHOS) and fatty acid oxidation (FAO) pathways [171, 185, 186]. OXPHOS upregulation is most commonly associated with a decrease in glycolysis. Activation of glycolysis can induce tumor cells to exit dormancy [187, 188]. Notably, metabolic organotropism may direct the metabolic adaptation of DCCs. For instance, DCCs in the liver rely on glycolytic metabolism, whereas those in the lung or bone rely on oxidative phosphorylation [170]. Upon targeted therapy, residual dormant cancer cells activate FAO and NRF2, a mediator of metabolic reprogramming essential for tumor recurrence [171]. Moreover, the NRF2-driven metabolic state renders residual cancer cells sensitive to glutaminase inhibition. Further, autophagy and mTOR activation in dormant cells may contribute to metabolic fitness and be responsible for maintaining dormancy [187, 189]. Similar to adult and embryonic stem cells, quiescent DCCs are expected to have a low rate of protein synthesis, ribosome biogenesis, translation, and RNA processing, which is correlated with their low cellular metabolism [190]. However, more investigations are needed to elucidate the mechanism and regulation of protein synthesis during DCC dormancy.

**4. Immune evasion**

In certain types of cancer, innate immune surveillance restricts the progression of dormant DCCs into fully developed metastases. For example, NK cells eliminate DCCs that re-enter the cell cycle and upregulate NK cell-activating ligands while downregulating MHCI [174, 191]. In other cancer types, the interferon gamma-driven NK cell cytostatic effect favors DCC dormancy [192]. The role of the adaptive immune system during dormancy has been described in several scenarios. In a uveal melanoma mouse model, CD8 + T cells favored the dormancy of DCCs at distant sites [35]. Likewise, in a pancreatic cancer mouse model, CD4 + T cells promoted the dormancy of DCCs in the presence of TNFR1 or IFNg signaling [193]. The cytotoxic effect of T cells could also be evaded through intrinsic signals from dormant DCCs. For instance, dormant, persistent acute leukemic cells resist cytotoxic T-cell-mediated lysis via the overexpression of B7-H1 or B7.1 [194]. Pancreatic dormant DCCs were shown to evade T cell immunity via MHCI downregulation [53].

**5. Survival mechanisms**

Quiescent dormant cancer cells escape antiproliferative drug-induced cell death [195, 196] because they are cell cycle arrested; however, new evidence suggest that the microenvironment also plays an important role in protecting them from these therapies [197]. Once at a new microenvironment, dormant cancer cells activate p38 MAPK signaling to survive the stress-induced niche [198–200]. PERK-elF2, BiP and ATF6 signaling are induced in dormant cells downstream of p38 signaling and regulate survival and quiescence phase [189, 201, 202]. Pancreatic dormant DCCs found in the liver of animals and human sections lack IRE1alpha activation and the spliced form of XBP1, but maintain phosphorylation of PERK [53]. Autophagy can also be induced in dormant cancer cells to survive upon targeted-therapy [203, 204]. Combination of Src inhibition together with MEK1/2 inhibition induces apoptosis of dormant breast cancer cells [200].

BOX 2-new classification of DCIS based on imaging biomarker systems Early diagnosis of breast and other cancers has improved significantly since the establishment of national and regional screening programs [205, 206]. For example, awareness and mammography screening are estimated to have reduced mortality from breast cancer by 20% in women aged 50–70 [207]. Additionally, studies have indicated that women participating in an organized mammography screening program experienced a 60% reduction in the risk of dying from breast cancer 10 years after diagnosis [208]. Similar successful examples can be observed in gastric cancer screening [209]. Nevertheless, with the implementation of these programs, overdiagnosis and concomitant overtreatment of patients also became evident [210]. For example, the breast pathology ductal carcinoma in situ (DCIS) has an estimated risk of death of only 3.3% [19]; however, it is estimated that 60% of women with this condition are treated with lumpectomy followed by radiation [211].

Attempts to distinguish high-risk patients (so they can receive the appropriate treatment) from low-risk patients (who would not experience benefit from treatment) include precision medicine [212], the implementation of multigene assays such as Oncotype DX or DCISion RT [213–215], or the identification of new and specific markers, including microRNAs [216, 217]. Some researchers have proposed that the factorization of radiographical images, originally designed for their screening value alone, could be useful for stratifying patients [218–224]. For example, a prospective randomized controlled mammography screening spanning four decades of follow-up, including nonpalpable lesions detected by imaging (mammography and MRI) and their correlation with corresponding histopathological features (from large histological sections), redescribed DCIS to be either derived from the terminal ductal lobular units (TDLU) or the lactiferous ducts. TDLU-derived lesions were described by imaging as having a crushed stone-like calcification when in situ and a stellated or circular oval when invasive. Simultaneously with the classification of acinar adenocarcinomas of the prostate and parotid glands [225, 226], the authors propose relabeling these lesions as *acinar adenocarcinomas of the breast* (AAB) [227], which are characterized by > 90% cumulative survival in a 25-year period [225]. Conversely, lesions with apparent origin within the major lactiferous ducts (relabeled by the same authors as ductal adenocarcinoma of the breast, DAB), with eight proposed subtypes, present around 60% overall survival within a 15-year follow-up period [228, 229]. Whether this controversial new classification [230] is accepted by the medical community is still unclear. However, it seems apparent that radiological images can become instrumental in stratifying patients based on the risk of severe disease, thus reducing overtreatment. It is intriguing to consider whether these different origins of DCIS can predict early dissemination in patients diagnosed with DCIS.
